# Saurian-associated *Leishmania tarentolae* in dogs: Infectivity and immunogenicity evaluation in the canine model

**DOI:** 10.1371/journal.ppat.1012598

**Published:** 2024-10-09

**Authors:** Jairo Alfonso Mendoza-Roldan, Ilaria Varotto-Boccazzi, Viviane Noll Louzada-Flores, Alec Evans, Imad Bouzaidi Cheikhi, Mariaelisa Carbonara, Andrea Zatelli, Sara Epis, Claudio Bandi, Frédéric Beugnet, Domenico Otranto

**Affiliations:** 1 Department of Veterinary Medicine, University of Bari, Valenzano, Italy; 2 Department of Biosciences, University of Milan, Milan, Italy; 3 Pediatric CRC ’Fondazione Romeo ed Enrica Invernizzi’, University of Milan, Milan, Italy; 4 Clinvet SA, Mohammedia, Morocco; 5 Boehringer Ingelheim Animal Health, Lyon, France; 6 Department of Veterinary Clinical Sciences, City University of Hong Kong, Hong Kong, SAR China; U.S. Food and Drug Administration and Center for Biologics Evaluation and Research, UNITED STATES OF AMERICA

## Abstract

In canine leishmaniosis endemic areas, *Leishmania infantum* may occur in sympatry with the non-pathogenic *Leishmania tarentolae*, which is associated to reptiles. The potential infectivity of *L*. *tarentolae* for mammals raises questions about the interactions between the two *Leishmania* species, and the potential cross-immune protection in dogs. This study aimed to assess the outcome of experimental *L*. *tarentolae* infection in dogs, determining: i) the anti-*L*. *tarentolae* antibody production, ii) the duration of the immunity and cytokine expression, and iii) the possible pathogenic effect in the canine host. Twelve purpose-bred beagle dogs were randomly allocated to three groups (intravenous inoculation, G1; intradermal inoculation, G2; negative control, G3). G1 and G2 dogs were inoculated twice (day 0, day 28) with 10^8^ promastigotes of *L*. *tarentolae* strain (RTAR/IT/21/RI-325) isolated from a *Tarentola mauritanica* gecko. The animals were followed until day 206. Blood, serum, conjunctival swabs and lymph node aspirate samples were collected monthly and bone marrow, liver and spleen biopsies on day 91. Hematological and biochemical parameters were assessed monthly, as well as serology (IFAT and ELISA) and molecular identification of *L*. *tarentolae*. Mononuclear cells (PBMC) were obtained to assess the cytokine expression through *in vitro* stimulation or (re-) infection. Data from this study demonstrated that DNA from *L*. *tarentolae* is detectable up to 3 months post-infection, with seroconversion after day 28. Moreover, the non-pathogenic nature of *L*. *tarentolae* was confirmed, with a neutral Th1/Th2 polarization, and a possible shift to Th1 phenotype after derived macrophages (re-) infection, as demonstrated by the expression of IFN-gamma. Therefore, *L*. *tarentolae* demonstrated a great potential as a surrogate pathogen and/or immune-prophylaxis/immune-therapy against *Leishmania* infections in dogs and humans.

## Author summary

The reptilian-associated *Leishmania tarentolae* may be infective for mammals, which raises questions about a potential cross-immune protection towards *Leishmania infantum* in dogs. This study aimed to assess the outcome of experimental *L*. *tarentolae* infection in the canine host. Twelve purpose-bred beagle dogs were allocated into three groups (intravenous inoculation, G1; intradermal inoculation, G2; negative control, G3). The first two groups were inoculated twice (day 0, day 28) with 10^8^ promastigotes of *L*. *tarentolae*, and were followed until day 206. Hematological and biochemical parameters were assessed, as well as serology (IFAT and ELISA), molecular identification and cytokine expression. Results demonstrated that DNA from *L*. *tarentolae* is detectable up to 3 months post-infection, with seroconversion after day 28. Moreover, the non-pathogenic nature of *L*. *tarentolae* was confirmed, with a neutral Th1/Th2 polarization, and a possible shift to Th1 phenotype (i.e., expression of IFN-gamma). These results suggest that *L*. *tarentolae* could be a potential candidate for developing immune strategies, opening new avenues for the prevention of the canine leishmaniasis.

## Introduction

Canine leishmaniosis (CanL) caused by *Leishmania infantum* is widespread in Southern Europe, Northern Africa, the Middle East, and the Americas [1]. In addition to dogs, this disease can affect humans [2]. Two main genera of sand flies are involved in the transmission of CanL, *Phlebotomus* in Africa and Eurasia, and *Lutzomyia* in the Americas [3–4]. In the Mediterranean Basin, *L*. *infantum* occurs in sympatry with primarily anthroponotic species of *Leishmania* (i.e., *Leishmania major* and *Leishmania tropica* in Northern Africa and the Middle East), and the reptile-associated *Leishmania tarentolae* [5–6]. The latter, which is included in the subgenus *Sauroleishmania*, occurs throughout the Mediterranean Basin, and is transmitted by herpetophilic sand fly species, such as *Sergentomyia minuta* [7–8]. Although *L*. *tarentolae* is considered non-pathogenic, even to its typical reptilian hosts (e.g., Moorish gecko, *Tarentola mauritanica*), transient infection of mammalian macrophages has been demonstrated *in vitro*, using some reference strains (e.g., the strain LEM-125) [6,9]. In addition, *L*. *tarentolae* might also infect mammals under natural conditions [6], as shown by molecular and serological positivity in humans and dogs in Italy [10–12]. The high abundance of *S*. *minuta* in leishmaniosis endemic areas [10,13], coupled with the behavior of this sand fly species, which may also feed on humans and dogs [14], supports the possibility that mammalian hosts may be exposed to *L*. *tarentolae*. In a nutshell, the sympatric occurrence of *L*. *infantum* and *L*. *tarentolae*, and the possibility that the latter might also infect dogs and humans, raises questions on the biological interactions between the two *Leishmania* species in areas endemic for CanL, including the potential cross-immune protection in the canine host [12]. This is coherent with current knowledge on the immunology of *L*. *tarentolae* in mammalian host models [9], and with the evidence for a lower level of anti-*L*. *infantum* antibody titers in clinically healthy sheltered dogs exposed to *L*. *tarentolae* [15–16].

The cross-protective immunity in *Leishmania* spp. infections has been demonstrated in other studies, for example using antigens of *Leishmania braziliensis* to protect against *L*. *infantum* infection [17–18]. Thus, co-infections of *L*. *tarentolae* and *L*. *infantum* may elicit natural protective immunity, suggesting *L*. *tarentolae* as a surrogate pathogen and potential vaccine candidate against CanL [6,9]. The use of *L*. *tarentolae* as a vaccine candidate would also be supported by the non-pathogenicity of this species in mammals [19]. In addition, it is worthy to emphasize that *L*. *tarentolae* is phagocyted and persists in dendritic cells and macrophages, until the formation of an amastigote-like state [20–21]. In the above experimental conditions, a slight shift toward the M1/Th1 phenotype has also been observed [22]. Nonetheless, despite the above evidence, knowledge gaps remain regarding the type of infection (e.g., transient or persistent), safety and pathogenicity in dogs, tissue tropism, also considering that reptiles do not have lymph nodes [23], and type of immunomodulation this protozoon determines in mammals. As a matter of fact, no experimental studies have been conducted yet in order to explore all the aspects above at once.

This study used a recently isolated strain of *L*. *tarentolae* from a gecko collected in Italy [13] to assess whether this species can infect dogs in an experimental setting and to determine: i) the anti-*L*. *tarentolae* antibody production, ii) the duration of the immunity and cytokine expression, and iii) the possible pathogenic effect in the canine host.

## Results

**Fig 1:**
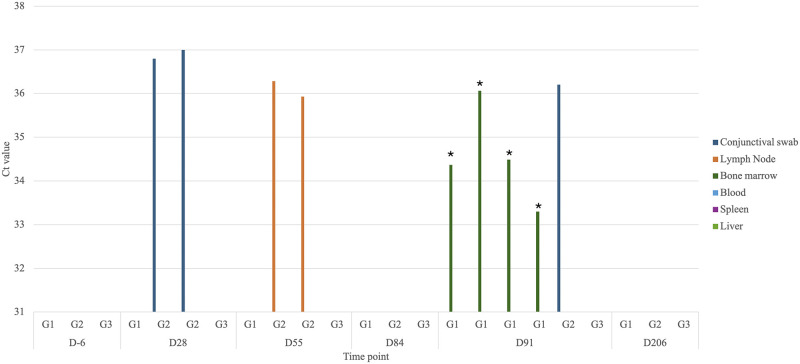
Molecular detection and quantitation cycle (Cq) values of *Leishmania tarentolae* in different tissues and timepoints. Asterisks (*) represent obtained sequences for ITS1 gene.

Eleven experimentally immunized dogs were monitored for 9 months, one dog from the G2 was removed from the study due to neurological signs not related to the infection. At the time of inoculation, the animals weighed 9–12 kg. *Leishmania tarentolae* was molecularly detected in four animals from G1 in bone marrow (day 91) and in four animals from G2, two in lymph nodes (day 55), and two in conjunctival swabs (day 28, 84 and 91) (Fig 1).

In addition, ITS1 sequences of *L*. *tarentolae* (blast homology 100% with *L*. *tarentolae* MT416149 from Italy) were obtained from the four positive animals in bone marrow. Nevertheless, *L*. *tarentolae* could not be cultured from bone marrow, liver or spleen biopsies. Dogs from G3 were negative in all the assessed time points.

**Fig 2:**
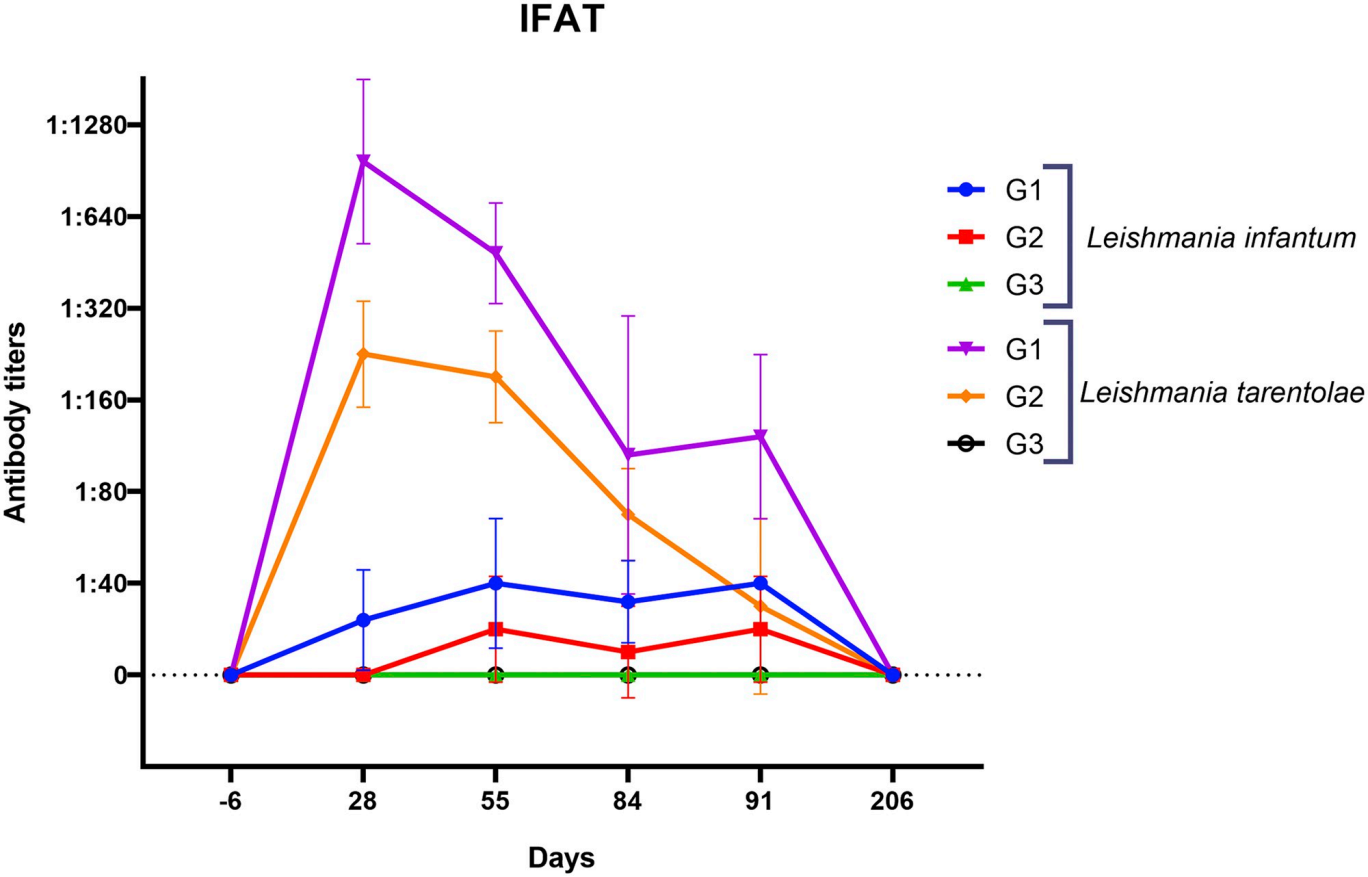
Anti-*Leishmania tarentolae* and *Leishmania infantum* antibody titers at indirect fluorescence immunoassay (IFAT). Results are shown according to different groups of dogs (G1 intravenous, G2 intradermic and G3 control) and time points.

At IFAT, all nine animals from G1 and G2 had detectable anti*-L*. *tarentolae* antibody titers from day 28, persistent until day 91 in 7 dogs (Fig 2), whereas dogs from G3 remained negative at all time points. In particular, G1 dogs had higher antibody titers at day 28 (1,1280), and remained positive until day 91, while dogs from G2 had titers up to 1:320 on day 28 and only two remained positive till day 91 (Fig 2). Conversely, 6 out of 9 dogs from G1 and G2 had 1:40 titers against *L*. *infantum* from day 55 (Fig 2). All dogs were negative at all time points for the commercial anti-*L*. *infantum* antibodies ELISA.

**Fig 3:**
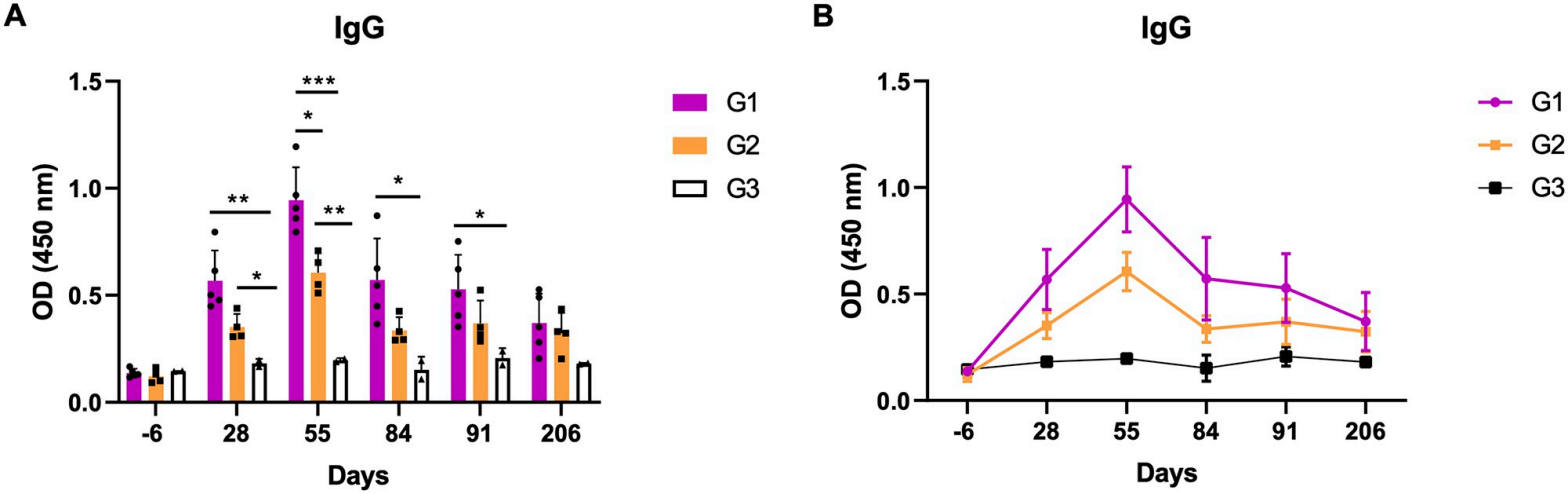
Specific anti-*Leishmania tarentolae* IgG antibody levels, detected by in-house enzyme-linked immunosorbent assay (ELISA). Each bar represents mean ± standard deviation. (A) Asterisk indicates the significant difference as determined after Tukey’s multiple comparisons test (p<0.05 denoted as *, p<0.01 denoted as **, p<0.001 denoted as ***). Dots represent original data points; (B) Symbols indicate mean ± standard deviation.

Levels of IgG antibody response were evaluated through in-house ELISA against *L*. *tarentolae* antigens (Fig 3). The analysis showed detectable levels of IgG antibodies in both G1 and G2 at all time points with statistically significant differences of both groups compared to the control group (day 28: *p*-values = 0.0067 and 0.0173; day 55: *p*-values = 0.0008 and 0.0052; day 84: only for G1 = 0.0172; day 91 only for G1: *p*-value = 0.0228). The levels of IgG antibodies decrease over time, with the lowest OD values after 206 days (Fig 3). The animals from G1 showed higher antibody levels compared to those from G2, yet the difference between the two groups was statistically different only at day 55 (*p*-value = 0.012).

Until day 206, none of the dogs presented clinical signs of disease, nor hematological or biochemically relevant alterations. Skin biopsies of dogs from G2 were all apparently normal and amastigotes were not observed. During the trial, the dogs in all groups maintained a good state of health (i.e., clinical score = 0), except for one G2 dog which presented a slightly higher clinical score of 2 (i.e., showing unrelated neurological signs) and was discharged.

**Fig 4:**
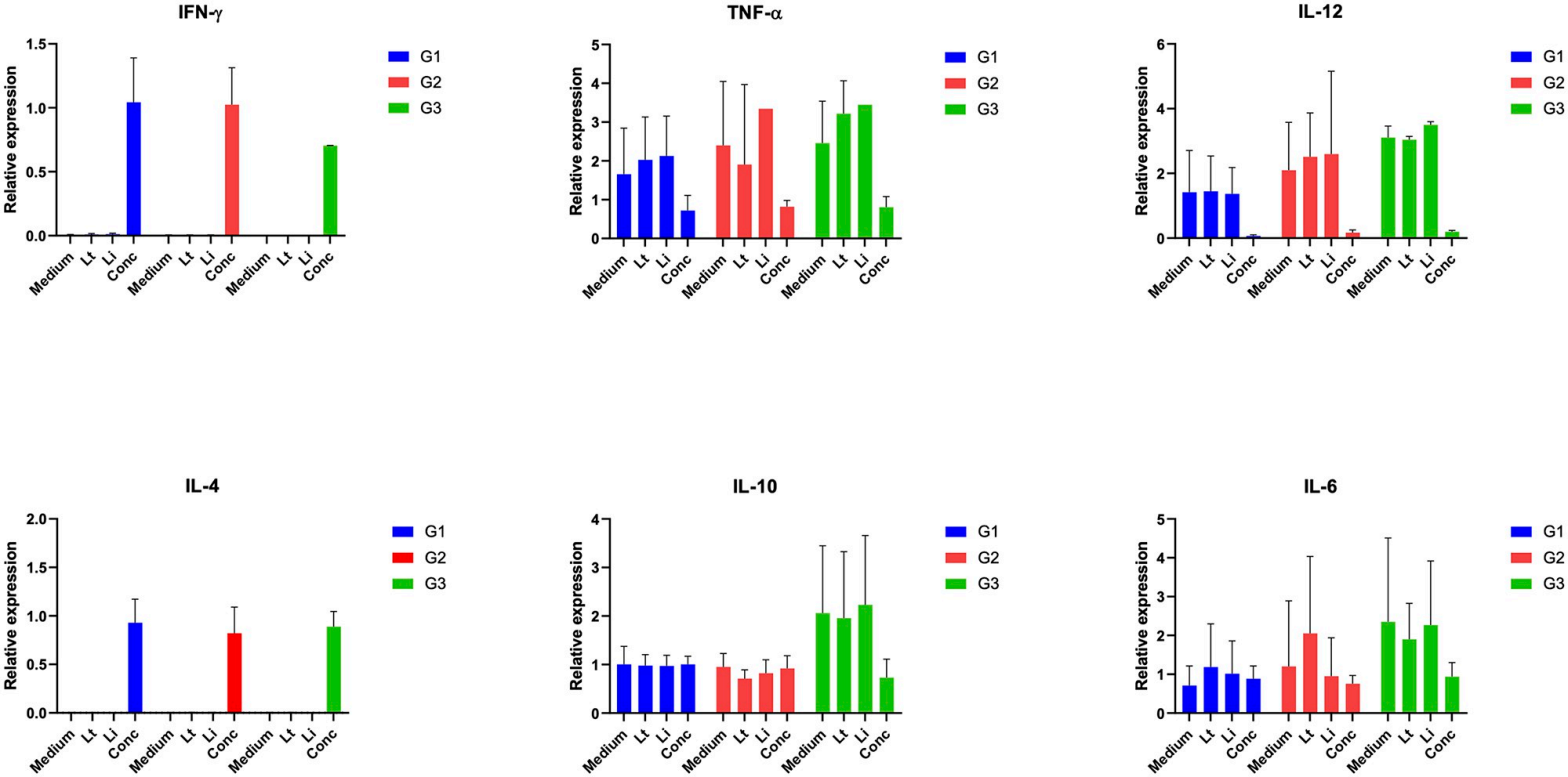
Relative gene expression of cytokines in peripheral blood mononuclear cells (PBMC). Stimulation after 24h with *Leishmania* spp. soluble antigen (LSA) of *Leishmania tarentolae* (Lt) or *Leishmania infantum* (Li), or with Concanavalin (Conc; positive control) or unstimulated (medium). Each bar represents mean ± standard deviation.

**Fig 5:**
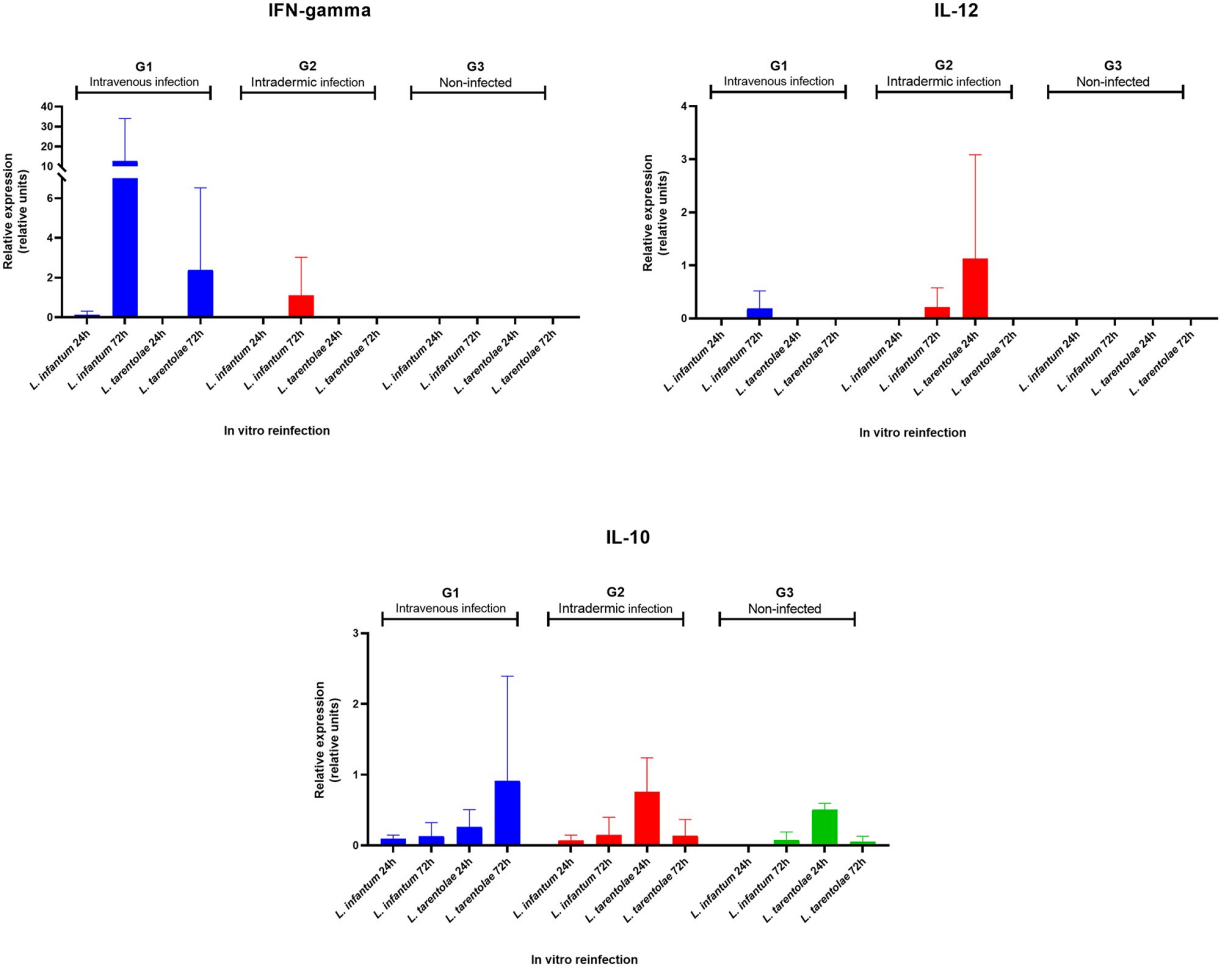
Relative gene expression (relative units) of pro-inflammatory cytokines IFN-gamma and IL-12 and anti-inflammatory cytokine IL-10. Data are shown according to each *in vitro* re-infection condition on G1 (intravenous), G2 (intradermic) and G3 (control) (standard deviation shown).

By stimulating the PBMC with lysate antigens, there was no evidence of either pro-inflammatory/Th1 response in infected dogs, or Th2 polarization (Fig 4). A slight decrease in IL-10 production was observed in G1 and G2 compared to G3 group (*p*-value = 0.0031 and *p*-value = 0.0014, respectively). TNF-α was produced in G1 and G2, but not significantly compared to controls. In G1, G2 and G3 dogs, both the IFN-gamma and IL-4 were not expressed. By *in vitro* (re-) infection of primary canine monocyte-derived mononuclear cells, pro-inflammatory cytokine IL-12 was expressed in G1 after 72h of infection with *L*. *infantum* (i.e., 0.57 relative units) and in G2 after 24h of infection with *L*. *tarentolae* (i.e., 3.387 relative units). The expression of IFN-gamma on G1 was observed mainly at 72h for *L*. *infantum* (i.e., 37.27 relative units) and for *L*. *tarentolae* (i.e., 7.16 relative units). This cytokine was detected in G2 after 72h of the *in vitro* infection with *L*. *infantum* (i.e., 3.32 relative units). No expression of TNF-α was detected. The expression of IL-10 in G1 with *L*. *tarentolae* re-infection at 72h was up to 2.621 relative units, while in G2 the expression of IL-10 reached 0.871 relative units with *L*. *tarentolae* re-infection at 24h. The expression of IL-10 in the G3 was higher at 24h with *L*. *tarentolae* infection (i.e., 0.570 relative units). In all groups, no expression of the anti-inflammatory cytokines IL-4 and IL-6 was observed after 72h of (re-) infection (Fig 5).

## Discussion

Data from this study demonstrated that DNA from *L*. *tarentolae* is detectable in experimentally infected dogs up to 3 months post-infection. In addition, at day 28 after the first inoculation, dogs seroconverted towards *L*. *tarentolae*. The simplest interpretation of these results is that *L*. *tarentolae* is capable of infecting dogs, at least with a survival of a few days or weeks and, possibly, with a few replication cycles. This is inferred by the DNA persistence in the host, up to the third month. On the other hand, animals did not demonstrate any clinical alterations after two doses of *L*. *tarentolae*. This confirms the non-pathogenic nature of this parasite and further validates the potential use of this protozoan as a surrogate agent for protecting dogs against CanL. As for the immunomodulation associated with this parasite, results are coherent with those of previous studies in murine and cellular models, indicating that *L*. *tarentolae* might be rather neutral in terms of Th1/Th2 polarization, perhaps with a possible shift on the Th1 phenotype, as indicated by the outcomes of experiments in macrophages *in vivo* [24].

The detection of *L*. *tarentolae* DNA after 90 days post-infection in the canine model is unprecedented. Indeed, data from studies using *in vitro* or murine models had previously indicated that *L*. *tarentolae* enticed a transient infection, presenting amastigote-like forms in PBMCs for few days [21,25]. Moreover, previous studies using primary canine PBMCs infected with recently isolated strains of *L*. *tarentolae* showed that this saurian-associated species can infect these cells, with a persistence of at least 5 days [21]. Conversely, when using laboratory strains of *L*. *tarentolae* that had been maintained in culture for several generations, the infection was not achieved even in a natural reptile host, the gecko *Hemidactylus turcicus* [26]. This suggests that lab strains that have been maintained *in vitro* for several generations have likely undergone genetic alterations, becoming incapable of inducing an infection *in vivo*. Therefore, in experimental studies, recently isolated strains of *L*. *tarentolae* should be used.

In addition, the results suggest that *L*. *tarentolae* can persist in canine hosts for longer periods of time than previously thought. However, as isolation of *L*. *tarentolae* was not possible, hence requiring further investigations for isolating this species from mammalian hosts in endemic areas.

On the other hand, the detection of *L*. *tarentolae* DNA from different tissues in experimentally infected dogs provided hints on the tissue tropism and life cycle of this parasite in the mammalian hosts, as well as the tissue samples to be examined in epidemiological studies of *L*. *tarentolae* in mammals. The molecular positivity in bone marrow of dogs from G1 is coherent with the idea that bone marrow is the most appropriate tissue for the molecular detection of *Leishmania* spp. [27]. Contrarywise, the molecular detection of *L*. *tarentolae* DNA in lymph nodes from dogs of G1 is unexpected, considering that reptiles do not have lymph nodes [28]. Yet, this last finding highlights that infection by *L*. *tarentolae* in mammals may follow a similar pathway to that observed in *L*. *infantum* infection in dogs. In addition, *in vitro* experiments, showing that *L*. *tarentolae* can infect mammalian macrophages and dendritic cells, is coherent with the possibility that this parasite could establish, at least for a short period, in lymph nodes. Finally, the molecular positivity in conjunctival swabs might offer a less invasive modality of sampling, appliable in future epidemiological studies for *L*. *tarentolae*, despite the fact that conjunctival swabs may have less sensitive diagnostic and prognostic performances, compared to other tissues tested for the detection of *L*. *infantum* [29].

Seroconversion at day 28 in G1 and G2 dogs demonstrated the immunogenicity of *L*. *tarentolae*, whereas in previous studies six out of thirteen dogs only seroconverted after 90 days of experimental infection with *L*. *infantum* [30]. Moreover, the cross-reactivity of some animals in IFAT for both species of *Leishmania* confirmed the diagnostic hindrance of this test, as previously observed in field studies [12]. Yet, the negativity of all dogs using the commercial ELISA for the detection of anti-*L*. *infantum* antibodies proved that animals were not co-infected and highlighted the usefulness of ELISA when using specific antigens, as demonstrated in comparative studies [16]. Indeed, the in-house ELISA using *L*. *tarentolae* antigens showed to be specific and sensitive revealing positivity even at day 206. Thus, future efforts are advocated to standardize a specific serological tool, such as ELISA, to better distinguish the infecting *Leishmania* species in endemic areas where both species occur.

Regarding immunological and cytokine responses, the results reported here are coherent with a previous study [31], in which the stimulation of human-derived dendritic cells with live *L*. *tarentolae* determined only moderate variation in the expression/release of Th1/Th2-associated markers. Indeed, the decrease in IL-10 cytokine herein observed in G1 and G2 might be due to a reduction of the anti-inflammatory feedback. Furthermore, the expression level of IL-12 (i.e., Th1 marker) and of IL-6 (i.e., pro-inflammatory marker) in inoculated dogs are comparable with the results obtained in control dogs. However, the *in vitro* re-infection with *L*. *tarentolae* or *L*. *infantum* on PBMC isolated from dogs experimentally infected with *L*. *tarentolae* (G1 and G2), highlights the potential protective effect that this non-pathogenic species may exert on macrophage cells, in terms of the expression of Th1 cytokines, that are normally associated with a protective immune response in CanL. Indeed, macrophages from dogs that had previously been infected with *L*. *tarentolae* displayed production of pro-inflammatory cytokines IFN-gamma and IL-12, after infection with *L*. *infantum*. Furthermore, the absence of the anti-inflammatory cytokines IL-4 and IL-6 expression further highlights that, after 72h of *L*. *infantum* infection, the cells did not produce inflammatory response. The molecular, serological and cytokine expression data obtained herein, coupled with the clinical evaluation of dogs (i.e., physical examination, hematological and urinary examinations) demonstrated the non-pathogenic nature of *L*. *tarentolae* in the canine host. Thus, this study validates the idea that *L*. *tarentolae* does not cause any clinical manifestation or clinical pathological alterations in dogs, despite having a similar behavior to that of *L*. *infantum*, with development of amastigote-like forms in mononuclear cells [9,32]. The basis for the non-pathogenicity of *L*. *tarentolae* is still not well understood. Indeed, this saurian-associated species possesses and expresses most of the virulence genes that are present in *L*. *major* and *L*. *infantum* [33], which indicates that the non-pathogenicity of *L*. *tarentolae* might derive from more complex factors than the simple absence of a gene. Accordingly, other studies showed that *L*. *tarentolae* present a deficiency of lipophosphoglycan (LPG) and proteases, as well as discrepancies in the presence and abundance of metabolites, compared to pathogenic species, where most of these metabolites are known to play a pivotal role in metacyclogenesis, infectivity, and ability to proliferate within the host cell [34]. Moreover, the comparison of the complete genome of a reference strain of *L*. *tarentolae* (*Parrott-TarII*) with other *Leishmania* species showed a similarity of over 90% of the gene content, yet it also indicated that *L*. *tarentolae* is better fitted to live as promastigote [19]. However, all the above studies on the genomics and biology of *L*. *tarentolae* should now be repeated using newly isolated strains, to avoid biases caused by the genetic modifications associated with long-term maintenance in cultures. Nonetheless, data herein reported regarding the non-pathogenicity of *L*. *tarentolae* in dogs confirms that *L*. *tarentolae* holds a great potential as a live vaccine against *L*. *infantum*, or as an immune- modulating agent to prevent or cure CanL.

In conclusion, data herein further suggests that *L*. *tarentolae* may infect and persist in the canine host, being also highly immunogenic, inducing antibody production. In addition, despite not having an evident shift towards the M1/Th1 profile in terms of cytokine expression, *in vitro* infection with *L*. *infantum*, in macrophage cells from dogs previously infected with *L*. *tarentolae*, displayed a Th1 response, that is typically associated with protection in most forms of leishmaniases. Importantly, results from this study are coherent with previous evidence of the infectivity of *L*. *tarentolae* in the dog host, with cross-reactivity in IFAT of *L*. *tarentolae* and *L*. *infantum*. Chiefly, the non-pathogenicity of *L*. *tarentolae* in the canine model is confirmed. Therefore, *L*. *tarentolae* holds great potential as a surrogate pathogen, to be used in vaccination or immune-prophylaxis/immune-therapy against infections by pathogenic *Leishmania* spp. in dogs and humans.

## Materials and methods

### Ethics statement

Animals were maintained according to the International Guiding Principles for Biomedical Research Involving animals in an insect proof environment to avoid any bite by sand flies or mosquitoes. The design and experimental procedures used in this study were authorized by the Clinvet Institutional Animal Care and Use Committee (Clinvet study n° CG1331-CVMO22/216). Moreover, this study was conducted in accordance with the principles of Good Clinical Practice (VICH GL9 GCP, 2000) adopted by the Committee for Medicinal Products for Veterinary Use (CVMP).

### Animals

A total of 12 purpose-bred beagles were enrolled in the study, conducted at Clinvet, Morocco. Animals were six months-old, deriving from two litters and previously dewormed and vaccinated against distemper, parvovirus, infectious hepatitis, leptospirosis and parainfluenza. Before infection, based on physical examination and laboratory test results (i.e., hemogram, complete biochemical profile, electrophoresis, urinalysis–see below), all animals were considered healthy. Moreover, the absence of previous exposure to *L*. *infantum* was confirmed by indirect fluorescence immunoassay (IFAT) and molecular analyses (see below).

### Source of parasites

*Leishmania tarentolae* strain (RTAR/IT/21/RI-325) isolated from a *T*. *mauritanica* gecko [6] and *L*. *infantum* strain (MCAN/IT/CRENAL/13903) isolated from a dog were used for the experimental procedures below. The strains were cryopreserved at −80 °C and cultures maintained at 26 °C for five days before infection, being cultivated in Schneider *Drosophila* medium supplemented with 10% of FBS (Fetal Bovine Serum), 0.1% of Penicillin-Streptomycin and 5% of human urine.

### Groups of animals and inoculation

**Fig 6:**
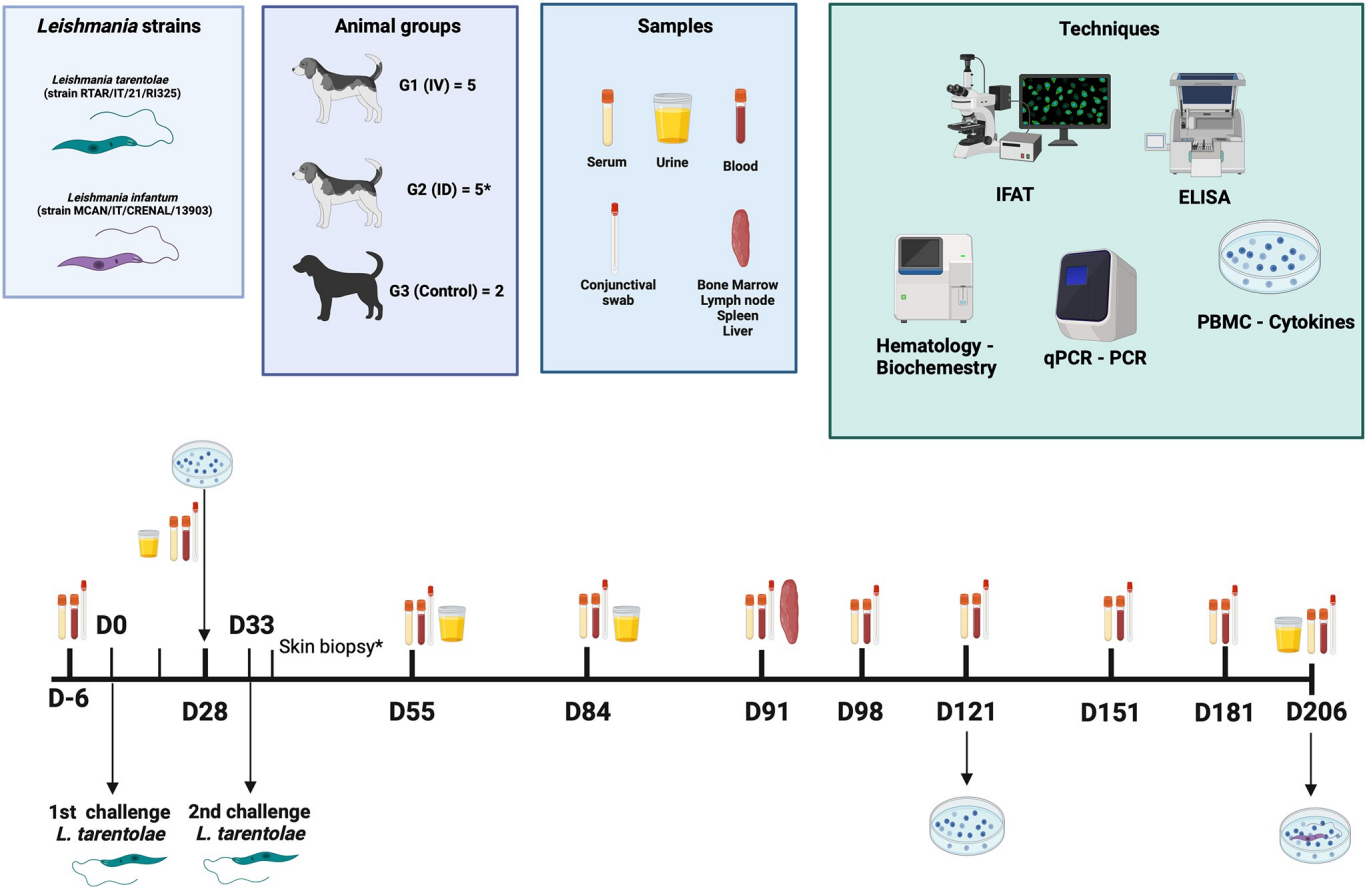
Study design of the experimental infection with *Leishmania tarentolae*. Routes of administration (G1 intravenous, G2 intradermic and G3 control), the type of samples and tissues collected, diagnostic techniques and cytokine expression tests employed. Created in BioRender.

Dogs were randomly allocated into three groups (intravenous inoculation, G1; intradermal inoculation, G2; negative control, G3). Animals in group G1 and G2, n = 5 animals each, were inoculated with 10^8^ promastigotes of *L*. *tarentolae* and two healthy dogs were used as negative control. Dogs in G1 received an administration of the *inoculum* in the cephalic vein, after catheter placement, whereas animals from G2 had an intradermal injection in the lateral surface of the neck. Dogs received in total two inoculations (i.e., day 0, day 28), after which animals were weekly monitored for the development of clinical signs using an updated clinical-based canine leishmaniosis scoring chart [28]. Monthly parasitological examination (molecular biology) was performed as well as hematobiochemical parameters, and serum antibody levels measured. Urine samples were collected and analyzed at days 28, 55, 85 and 206. Lymph node aspiration and conjunctival swabs were conducted monthly (see Fig 6), while liver, bone marrow and spleen biopsies were performed on day 91.

### Hematological parameters and urine examination

Blood samples were collected from either the cephalic or jugular veins into K3 EDTA tubes (2 ml) to undergo routine hematology, and in a plain tube (5 ml) to obtain serum after centrifugation (15 min at 1500 x g). For each enrolled dog, a complete blood count (CBC) with reticulocyte count, a complete biochemical panel, including acute phase proteins (i.e., CRP and ferritin) and sSDMA concentration measurement, and a serum capillary electrophoresis, and were performed.

Serum samples were stored at -20°C until analysis. Serum biochemical analysis (Beckman Coulter, Clinical Chemistry Analyzer AU680, Indianapolis, United States), and serum protein electrophoresis (SEBIA Italia S.r.l., Capillarys 2 Flex Piercing, Florence, Italy) were performed using the same methods in all tested samples. All urine samples were collected via ultrasound-guided cystocentesis in BD Vacutainer^®^ urinalysis preservative tubes (5 ml) and stored at -20°C until analysis. To determine the urine protein/creatinine (UPC) ratio, the protein concentration (mg/dl) was assessed using pyrogallol red-molybdate assay, while the serum creatinine (mg/dl) was measured through the Jaffé method in undiluted urine (Beckman Coulter, Clinical Chemistry Analyzer AU680, Indianapolis, United States).

### *Leishmania tarentolae* parasite burden determination

The presence of promastigotes in bone marrow, liver and spleen aspirates at day 91 was evaluated by Tobie-Evans medium cultures maintained at 26°C and monitored weekly over a 5-week period. Skin biopsies were performed 24 hours (h) after the second inoculation of *L*. *tarentolae* (day 33) in dogs from G2 through punch biopsies (2 mm), near the inoculation site on the neck, that were formalin-fixed and after histologically processed.

Genomic DNA (gDNA) was extracted from blood, lymph nodes, conjunctival swabs, liver, spleen, and bone marrow using two commercial kits, GenUP gDNA and GenUP Blood DNA kits (Biotechrabbit GmbH, Hennigsdorf, Germany), respectively, according to the manufacturer’s instructions. All samples were tested by duplex real-time PCR (dqPCR) for the detection of *L*. *infantum* and/or *L*. *tarentolae* and they were considered positive with quantitation cycle (Cq) values up to 38.0 and 38.6, respectively [35]. Approximately 100 ng of gDNA, with the exception of the no-template control, was added to each dqPCR. Genomic DNA from cultured promastigotes of *L*. *infantum* (zymodeme MON-1), and *L*. *tarentolae* were used as positive controls. Samples were also tested for *L*. *infantum* kDNA minicircle (120 bp) by real-time PCR (qPCR), using the protocol described elsewhere [36]. For sequence analyses, *Leishmania* dqPCR-positive samples were amplified by conventional PCR (cPCR) using primers L5.8S/LITSR targeting the partial region of the internal transcribed spacer 1 (ITS1, ~ 300 bp) and PCR protocol run as described elsewhere [37].

### Serological tests

Serum samples from each group of dogs were tested to assess the exposure to *L*. *infantum* and *L*. *tarentolae*. An IFAT for the detection of IgG anti-*L*. *infantum* was performed as previously described [38], whereas for antibodies against *L*. *tarentolae*, IFAT was performed using promastigotes of *L*. *tarentolae* as antigen, following the same procedure as for *L*. *infantum* IFAT described elsewhere [12]. For both IFAT above, serum samples from a dog positive for *L*. *infantum* by cytological and molecular analyses, and a healthy dog scoring negative for *L*. *infantum*, were used as positive and negative controls, respectively. Samples were scored as positive when presenting a clear cytoplasmic and membrane fluorescence of promastigotes from a cut-off dilution of 1:40, with positive samples serially titrated until negative results were obtained.

In addition, serum samples were tested for anti-*L*. *infantum* antibodies using a commercial enzyme-linked immunosorbent assay (ELISA) (VetLine Leishmania ELISA, Novatec Immunodiagnostica GmbH, Germany).

### Production of *Leishmania* spp. soluble antigen (LSA)

*Leishmania* soluble antigen (LSA) of *L*. *tarentolae* (P10 strain) and *L*. *infantum* (MHOM/TN/80/IPT1 strain) were prepared according to the protocol described previously [24]. Briefly, *Leishmania* cells were washed in PBS and lysed with a solution containing 50 mM Tris, 5 mM EDTA and protease inhibitors (Invitrogen, Waltham, MA, USA), followed by three rapid freeze/thaw cycles and six sonication pulses of 20s/40W. The supernatants were subsequently collected and the protein concentration was determined through the Nanodrop spectrophotometer.

### In-House *Leishmania* IgG ELISA

Specific *Leishmania* antibodies were detected in canine sera with an in-house IgG ELISA, using LSA of *L*. *tarentolae* as coating antigen. The ELISA was performed as described in previously [24]. Briefly, plates were coated with 3 μg/ml of *L*. *tarentolae* LSA and incubated overnight at 4°C. Plates were washed three times with tris-buffered saline (TBS)–0.05% (v/v) Tween 20 (TBS-T); then, they were blocked with TBS-T containing 5% (w/v) non-fat dry milk (NFDM; Euroclone, Pero, Italy) for 1h. Sera were two-fold serial diluted, starting from 1:50 in blocking buffer and incubated for 1h at 26°C. Then, goat anti-dog IgG HRP-conjugated (Abcam, Cambridge, UK) diluted at 1:50000 was added for 30 min at 26°C. Finally, 3,30,5,50-tetramethylbenzidine substrate (TMB) (Sigma Aldrich, St. Louis, MO, USA) was added for 20 min followed by HCl solution 0.5 N to stop the reaction (Fisher Chemical, Waltham, MA, USA).

### PBMC isolation

Mononuclear cells were obtained according to procedures previously described [21]. Briefly, peripheral blood mononuclear cells (PBMC) were isolated using lymphocyte separation medium (Cytiva, USA) from 10 ml of heparinized blood collected from 11 dogs. After two washes with warm PBS at 1500 rpm for 10 min, erythrocytes were removed with the erythrocyte lysis buffer (Euroclone, Pero, Italy). The cells were counted and adjusted up to 10^6^ cells/ml in warm RPMI-1640 medium (Euroclone, Milan, Italy) supplemented with 10% heat-inactivated fetal bovine serum and 1% Glutamine.

### PBMC stimulation using LSA

PBMC were incubated with either complete media (unstimulated), LSA of *L*. *infantum* (10 μg/ml) or LSA of *L*. *tarentolae* (10 μg/ml), or Concavalin A (ConA) (10 μg/ml) as positive control, in a 12-well plate at 37°C for 24 h in 5% CO2 atmosphere. At the end of incubation, PBMC were collected and stored in RNA later (RNAprotect^®^ Cell Reagent, Qiagen) at 4°C until use.

### *In vitro* (re-)infection of primary canine monocyte-derived mononuclear cells

From each group, a total of 10^6^ mononuclear cells/ml from three randomly selected dogs were seeded into 24-well plates and maintained at 37°C with 5% CO_2_ for 24 h for the adhesion of the cells. The medium was subsequently replaced by fresh RPMI-1640 supplemented with 10% of FBS and Penicillin-Streptomycin (100X). The plate was incubated for 120 h at 37°C with 5% CO_2_. A negative non-infected control was included in each experimental assay.

For the mononuclear phagocytic assays, *L*. *tarentolae* (RTAR/IT/21/RI-325), and *L*. *infantum* (MCAN/IT/CRENAL/13903) promastigotes at the stationary phase of growth were centrifuged at 3000 x *g* for 10 minutes (min) at room temperature and washed twice with sterile PBS (1X). The resulting pellet was resuspended in RPMI-1640, and the parasites were counted in a Neubauer chamber at a dilution ratio of 1:100. Subsequently, promastigotes at a concentration of 10^6^ parasites/ml (parasite/macrophage ratio 10:1) were seeded to the plate and incubated at 37°C with 5% CO_2_. After 4h of infection, the wells were washed twice with sterile PBS to remove non-internalized parasites. The plates were monitored during post-infection time intervals (24h and 72h). All experiments were conducted in duplicate and a negative non-(re-) infected control was added for the three groups.

### RNA extraction and quantitative reverse transcription PCR (RT-qPCR)

RNA extraction and RT-qPCR protocols were applied for both: i) collected PBMC stimulated with LSA and ii) primary canine monocyte-derived mononuclear cells. Scraped cells were collected at 24h, (48h and 72h for re-infection) after stimulation or infection and subjected to centrifugation at 12000 x *g* for 10 min at RT and the pellet was submitted to RNA extraction by AllPrep DNA/RNA/Protein Mini Kit or RNeasy^®^ Plus Mini Kit, Qiagen (Qiagen, USA) according to the manufacturer’s guidelines. The concentration of total RNA was quantified by Nanodrop and the purity was checked by determining the 260/280 nm absorbance ratio. RNA was stored at −80°C until use. The synthesis of cDNA was performed in 10 μl reverse transcription reactions using 50 ng of total RNA in 1X SuperScript IV VILO Mastermix (Thermo Fisher Scientific, Whaltam, USA) following the manufacturer guidelines. The cDNA concentration was quantified by Qubit dsDNA HS Assay Kit and stored at -20°C.

Quantitative PCR (qPCR) analyses were performed to determine the gene expression of IL-6, IL-10, IL-4 and IFN-gamma; TNF- α, IL-12 cytokines (S1 Table). The reactions were performed with 10 μl of SsoAdvanced^™^ Universal Supermix (Bio-Rad, CA, USA) and 0.7 μl of PrimerPCR Costum Assay of primers (400 nM) and probes (250 nM) [39] at 55°C for 2 min, 95°C for 10 min, followed by 45 cycles of 95°C for 15 sec and 60°C for 1 min. High-resolution melting curves of PCR amplicons were obtained with temperatures ranging from 65°C to 95°C with a 0.5°C increase in temperature every five seconds. For normalization purposes, two housekeeping genes (i.e., G3PDH, [39] and OAZ1, [40]) were included. The gene expression of each cytokine was evaluated by the method 2^-ΔΔCq^ value [41] and presented as mRNA relative units.

### Statistical information

Data were analyzed with two-way ANOVA, followed by Tukey’s multiple comparisons test for serological analyses or by Dunnett’s multiple comparison test for RT-qPCR analyses. Statistics were performed using GraphPad Prism 8.0 (GraphPad, CA, USA). The results were considered statistically significant when *p*-value was less than 0.05.

## Supporting information

S1 Table List of primer sequences. (PDF)
