# Supplementary material for: Saurian-associated Leishmania tarentolae in dogs: Infectivity and immunogenicity evaluation in the canine model
Source: PLoS Pathog. 2024 Oct 9;20(10):e1012598. doi: 10.1371/journal.ppat.1012598 (PMC11463833; doi:10.1371/journal.ppat.1012598)
Supplement: S1 Table — (PDF) [file ppat.1012598.s001.pdf]

Supplementary Table 1: List of primer sequences

**Gene**	**Product Length (pb)**	**Primer Forward**	**Primer Reverse**
*G3PDH**	90	TCAACGGATTTGGCCGTATTGG	TGAAGGGGTCATTGATGGCG
*OAZ1**	168	CGGCTGCCTCTACATCGAGA	AAGCTGAAGGTCCGGAGCAA
IL-4	123	GCTCCAAAGAACACAAGCGA	CATGCTGCTGAGGTTCCTGT
IL-6	102	CTCTCCACAAGCGCCTTCTC	TGAAGTGGCATCATCCTTGG
IL-10	101	CGACCCAGACATCAAGAACC	CACAGGGAAGAAATCGGTGA
IFN-gama	113	TCAACCCCTTCTCGCCACT	GCTGCCTACTTGGTCCCTGA
TNF-alfa	84	CTGGAGTCGTGAGGCAGTG	AGGGCTCTTGATGGCAGAGA
IL-12	109	CAGCAGAGAGGGTCAGAGTGG	ACGACCTCGATGGGTAGGC
